# Supplementary material for: Experimental search for high-temperature ferroelectric perovskites guided by two-step machine learning
Source: Nat Commun. 2018 Apr 26;9:1668. doi: 10.1038/s41467-018-03821-9 (PMC5920103; doi:10.1038/s41467-018-03821-9)
Supplement: Supplementary file 1 — Supplementary Information [file 41467_2018_3821_MOESM1_ESM.pdf]

Experimental search for high-temperature ferroelectric perovskites guided by two-step machine learning

Prasanna V. Balachandran,[1, a)] Benjamin Kowalski,[2] Alp Sehirlioglu,[2, b)] and Turab Lookman[1, c)]

[1)] *Theoretical Division, Los Alamos National Laboratory, Los Alamos, NM 87545, USA.*

[2)] *Department of Materials Science and Engineering, Case Western Reserve University, Cleveland, OH 44106, USA.*

(Dated: 28 February 2018)

[a)]Electronic mail: pvb5e@virginia.edu

[b)]Electronic mail: axs461@case.edu

[c)]Electronic mail: txl@lanl.gov

Supplementary Table 1 | Ionic displacements (δ, in Å) for the Me-cations listed in the table were taken from the work of Balachandran *et al*.[1] For Pb^{2+} and Bi^{3+}, the values for δ were taken to be 0.45 and 0.80 Å, respectively,[2] from the literature.

Me-cation	Ionic Displacements (δ, in Å)
Mg^{2+}	0.084
Ni^{2+}	0.081
Zn^{2+}	0.233
Fe^{3+}	0.169
In^{3+}	0.088
Mn^{3+}	0.131
Sc^{3+}	0.109
Yb^{3+}	0.081
Al^{3+}	0.109
Co^{3+}	0.178
Cr^{3+}	0.127
Ga^{3+}	0.095
Lu^{3+}	0.077
Ti^{4+}	0.169
Zr^{4+}	0.131
Hf^{4+}	0.129
Sn^{4+}	0.108
Nb^{5+}	0.169
Ta^{5+}	0.169
Sb^{5+}	0.132
Bi^{5+}	0.134
Mo^{6+}	0.107
W^{6+}	0.101

Supplementary Note 1

Our first iteration based on the recommendations from the classification learning and EGO method led to the $0.6Bi(Sc_{0.17}Ga_{0.83})O_3$–0.4PT composition and its predicted $\widehat{T_C}$ was equal to 747 ± 223 K. However, our XRD measurements did not reveal a pure perovskite phase. We augmented our classification learning dataset (shown as D1 in Figure 2 in the main manuscript) with this composition and assigned it a −1 label. We then retrained our SVC_{rbf} classifiers.

In the second iteration, the EGO method recommended $0.35Bi(In_{0.31}Sc_{0.69})O_3$–0.65PT, whose predicted $\widehat{T_C}$ was 923 ± 343 K. Our XRD measurements revealed a pure perovskite phase and we measured its T_C to be 733 K. During this step, we also considered compositions in the $Bi(ScGa)O_3$–PT pseudo-ternary phase space, which were unsuccessfully predicted by ML in the first iteration. The most promising composition, *i.e.,* the one down-selected by the SVC_{rbf} classifier and recommended by EGO, was identified as $0.5Bi(Sc_{0.56}Ga_{0.44})O_3$–0.5PT, whose predicted $\widehat{T_C}$ was 846 ± 229 K. Our XRD measurements revealed that this composition still contained secondary phases. We augmented our classification learning dataset with these two compositions, namely $0.35Bi(In_{0.31}Sc_{0.69})O_3$–0.65PT and $0.5Bi(Sc_{0.56}Ga_{0.44})O_3$–0.5PT with the labels +1 and −1, respectively. Our regression dataset (shown as D2 in Figure 2 in the main manuscript) was augmented with only the $0.35Bi(In_{0.31}Sc_{0.69})O_3$–0.65PT perovskite composition, whose measured T_C was 733 K. We retrained both SVC_{rbf} and SVR_{rbf} models with the inclusion of these new data points.

After the second iteration, Kowalski and Sehirlioglu[3] shared XRD and dielectric measurement results for 13 new compositions that were not originally present in our training datasets: 12 in the $BiScO_3$–$BiInO_3$–$PbTiO_3$ (the $BiInO_3$ content was < 15%) and one in the $BiScO_3$–$BiGaO_3$–$PbTiO_3$ composition space. The XRD measurements revealed that all $BiScO_3$–$BiInO_3$–$PbTiO_3$ compositions formed in the perovskite phase and we augmented the classification learning dataset with these compositions and assigned +1 label to each of them. Similarly, we also augmented the regression dataset with the T_C data for these 12 compositions. In the case of $BiScO_3$–$BiGaO_3$–$PbTiO_3$, XRD revealed secondary phases and therefore, it was assigned a −1 label. Note that none of these compositions were recommended by hierarchical learning. The XRD data for the 13 compositions and the dielectric measurements data for the 12 perovskite compositions are given in the Supplementary Figure 1.

In the third iteration, our EGO algorithm recommended $0.25Bi(Sc_{0.47}Ga_{0.53})O_3$–0.75PT whose $\widehat{T_C}$ was 778 ± 135 K. The XRD data revealed pure perovskite phase and we measure its T_C as 798 K, in excellent agreement with our predicted $\widehat{T_C}$ value. In the third iteration, we also synthesized the $0.7Bi(Fe_{0.73}Co_{0.27})O_3$–0.3PT composition, which had the maximum $\widehat{T_C}$ (929 ± 94 K) in the unexplored composition space. We identify this as an interesting composition, because the {FeCo} pair do not exist either in the classification learning or regression datasets. This pair is also not explored in the literature. However, the XRD data revealed presence of secondary phases. Thus, we augmented our classification learning dataset with $0.25Bi(Sc_{0.47}Ga_{0.53})O_3$–0.75PT and $0.7Bi(Fe_{0.73}Co_{0.27})O_3$–0.3PT and assigned +1 and −1 labels, respectively. We also augmented the regression dataset with $0.25Bi(Sc_{0.47}Ga_{0.53})O_3$–0.75PT, whose measured T_C was 798 K.

In the fourth iteration, the EGO algorithm recommended $0.4Bi(Fe_{0.19}Sc_{0.81})O_3$–0.6PT with a predicted $\widehat{T_C}$ of 749 ± 161 K. Similarly, the $0.2Bi(Fe_{0.12}Co_{0.88})O_3$–0.8PT solid solution had the maximum $\widehat{T_C}$ (815 ± 24 K) in the unexplored space. Our XRD measurements revealed that both compositions formed in the pure perovskite phase. The measured T_C for $0.4Bi(Fe_{0.19}Sc_{0.81})O_3$–0.6PT and $0.2Bi(Fe_{0.12}Co_{0.88})O_3$–0.8PT were found to be 728 and 898 K, respectively. We augmented our classification learning and regression datasets with +1 labels and T_C data, respectively, for both $0.4Bi(Fe_{0.19}Sc_{0.81})O_3$–0.6PT and $0.2Bi(Fe_{0.12}Co_{0.88})O_3$–0.8PT compositions.

In the fifth iteration, the top two recommendations from the EGO algorithm include, $0.3Bi(Yb_{0.44}Al_{0.56})O_3$–0.7PT and $0.3Bi(Ni_{0.50}Sn_{0.50})$O3–0.7PT and its predicted $\widehat{T_C}$ values were 801 ± 125 and 780 ± 123 K, respectively. In this iteration, we decided to study two compositions from EGO because they belonged to two novel {Me′Me″} pairs that are also not explored in the literature. On the other hand, $0.2Bi(Co_{0.90}Al_{0.10})O_3$–0.8PT had the maximum predicted $\widehat{T_C}$ value of 843 ± 33 K and the {CoAl} pair is also not explored in the literature. The XRD measurements revealed that the $0.3Bi(Yb_{0.44}Al_{0.56})O_3$–0.7PT had secondary phases. On the other hand, a pure perovskite phase was found in the $0.3Bi(Ni_{0.50}Sn_{0.50})$O3–0.7PT and $0.2Bi(Co_{0.90}Al_{0.10})O_3$–0.8PT compositions. The T_C data for $0.3Bi(Ni_{0.50}Sn_{0.50})O_3$–0.7PT and $0.2Bi(Co_{0.90}Al_{0.10})O_3$–0.8PT compositions were measured as 658 and 883 K, respectively. The XRD and dielectric measurements data are given in Figures 4 and 5 in the main manuscript.

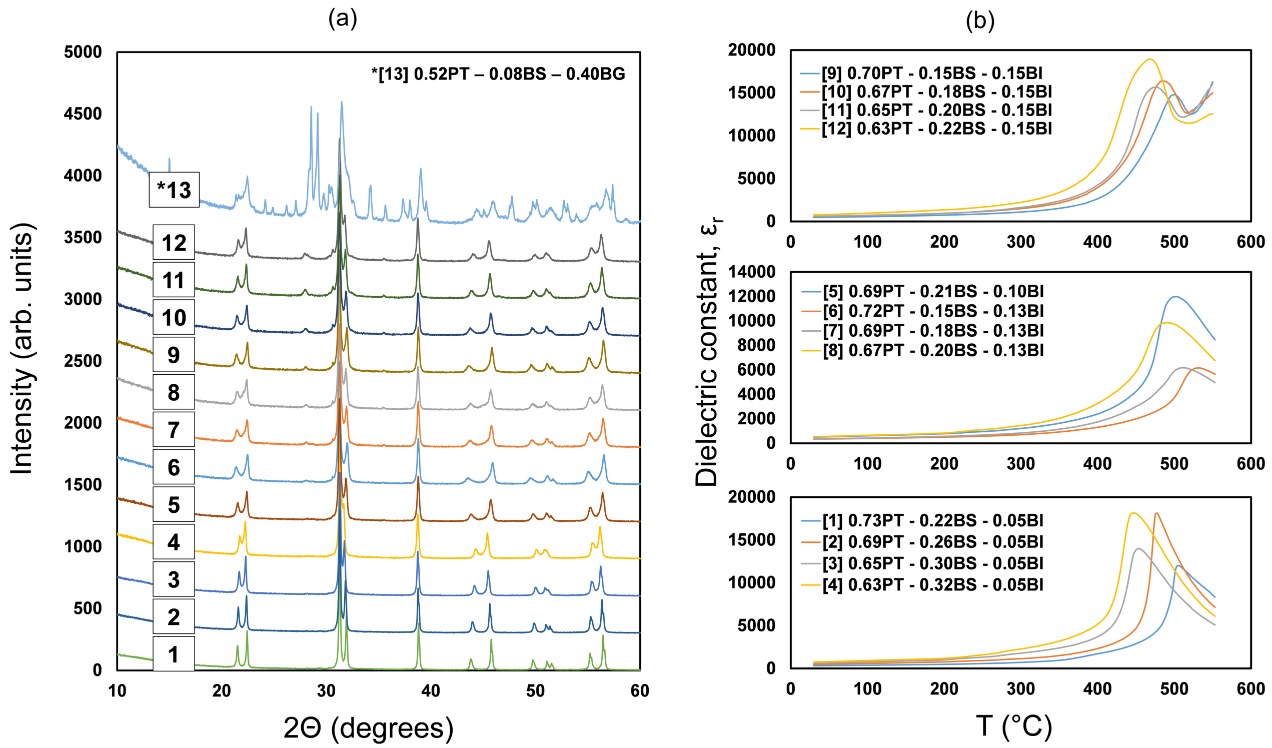

Supplementary Figure 1 | **X-ray diffraction and dielectric measurements data.** (a) XRD data for 13 compositions. Compositions 1 to 12 correspond to that of the $PbTiO_3$ (PT)-$BiScO_3$ (BS)-$BiInO_3$ (BI) system, whereas composition 13 corresponds to that of the PT-BS-$BiGaO_3$ (BG) pseudo-ternary space. All 12 PT-BS-BI compositions were identified to be perovskite, however the PT-BS-BG composition has secondary phases and therefore not labeled as a perovskite (identified with an asterisk symbol, $*$). (b) Dielectric data measured at 10 kHz frequency as a function of temperature (T, $^\circ$C) for the 12 PT-BS-BI compositions.

Supplementary References

[1]P. V. Balachandran, T. Shearman, J. Theiler, and T. Lookman, Acta. Cryst. B **73**, 962 (2017).
[2]I. Grinberg and A. M. Rappe, Phase Transitions **80**, 351 (2007).
[3]B. Kowalski and A. Sehirlioglu, Private Communication.
